# Neonatal and postneonatal tetanus at a referral hospital in Kamsar, Guinea: a retrospective audit of paediatric records (2014–2018)

**DOI:** 10.1093/inthealth/ihab021

**Published:** 2021-05-28

**Authors:** Ibrahima Condé, Mahamoud Sama Cherif, Prabin Dahal, Marie Elisabeth Hyjazi, Facely Camara, Macka Diaby, Abdoul Salam Diallo, Adeniyi Kolade Aderoba, Foumba Conde, Mohamed Lamine Diallo, Fatoumate Binta Diallo, Hasmiou Dia, Mamadou Pathé Diallo, Alexandre Delamou, Telly Sy

**Affiliations:** Faculty of Sciences and Health Technics, Gamal Abdel Nasser University of Conakry, Conakry, Guinea; Pediatrics Department Kamsar Hospital, Kamsar, Guinea; Faculty of Sciences and Health Technics, Gamal Abdel Nasser University of Conakry, Conakry, Guinea; Centre for Tropical Medicine and Global Health, University of Oxford, Oxford, UK; Faculty of Sciences and Health Technics, Gamal Abdel Nasser University of Conakry, Conakry, Guinea; Pediatrics Department Kamsar Hospital, Kamsar, Guinea; Faculty of Sciences and Health Technics, Gamal Abdel Nasser University of Conakry, Conakry, Guinea; Faculty of Sciences and Health Technics, Gamal Abdel Nasser University of Conakry, Conakry, Guinea; Pediatrics Department Kamsar Hospital, Kamsar, Guinea; Faculty of Sciences and Health Technics, Gamal Abdel Nasser University of Conakry, Conakry, Guinea; Pediatrics Department Kamsar Hospital, Kamsar, Guinea; National Perinatal Epidemiology Unit, University of Oxford, Oxford, UK; University of Medical Sciences Teaching Hospital, Akure, Nigeria; Pediatrics Department Kamsar Hospital, Kamsar, Guinea; Faculty of Sciences and Health Technics, Gamal Abdel Nasser University of Conakry, Conakry, Guinea; Faculty of Sciences and Health Technics, Gamal Abdel Nasser University of Conakry, Conakry, Guinea; Faculty of Sciences and Health Technics, Gamal Abdel Nasser University of Conakry, Conakry, Guinea; Faculty of Sciences and Health Technics, Gamal Abdel Nasser University of Conakry, Conakry, Guinea; Faculty of Sciences and Health Technics, Gamal Abdel Nasser University of Conakry, Conakry, Guinea; Faculty of Sciences and Health Technics, Gamal Abdel Nasser University of Conakry, Conakry, Guinea

**Keywords:** clinical audit, elimination, Guinea, neonates, tetanus, vaccination

## Abstract

**Background:**

Tetanus is a vaccine-preventable disease caused by the bacterium *Clostridium tetani*. In 2018, all of Guinea was considered to be at risk of the disease and the country is currently in the elimination phase.

**Methods:**

A 5-y audit (1 January 2014–31 December 2018) of all admissions to the neonatal and general paediatric units of Kamsar Hospital (Western Guinea) was undertaken to identify cases of neonatal tetanus (NNT) and postneonatal tetanus (PNNT).

**Results:**

There were 5670 admissions during the study period, of which 39 (0.7%) were due to tetanus (22 NNT and 17 PNNT). Among NNT patients, the bacterial entry site was the umbilical cord (n=20) or wound following circumcision (n=2). For PNNT, the entry site was surface wound (n=12), limb fracture (n=1) or could not be established (n=4). A majority of the patients (36/39, 92.3%) were born to unvaccinated mothers or those who received suboptimal vaccination during pregnancy. Overall, 21 (53.8%) children died within 7 d of admission with a higher mortality observed among neonates (16/22, 72.7%) compared with postneonates (5/17, 29.4%).

**Conclusions:**

Tetanus was a rare cause of admission at Kamsar Hospital with a very high case fatality rate. The disease primarily occurred among children born to mothers who were unvaccinated/inadequately vaccinated during pregnancy.

## Introduction

Tetanus is a vaccine-preventable disease caused by a neurotoxin produced by the bacterium *Clostridium tetani*. The global burden of tetanus has gradually declined from an estimated 800 000 to 1 million deaths in the 1980s and 1990s to 56 743 (95% confidence interval (CI): 48 199 to 80 042) in 2015.^1,2^ Despite this sharp decline, tetanus remains one of the top infectious causes of under-five mortality in West Africa.^3^ In particular, neonatal tetanus (NNT), defined as tetanus occurring within 28 d after birth, accounts for the majority of tetanus-related deaths.^4^ Newborns from unvaccinated mothers and those born at home are at an increased risk of exposure to the spores of the bacterium due to traditional umbilical cord practices such as the application of *Karité* nut butter, herbs or cow dung.^5–^^9^ Postneonatal tetanus (PNNT) occurring outside the neonatal period is less common and is associated with substantially lower mortality compared with NNT.^2^

**Figure 1.**
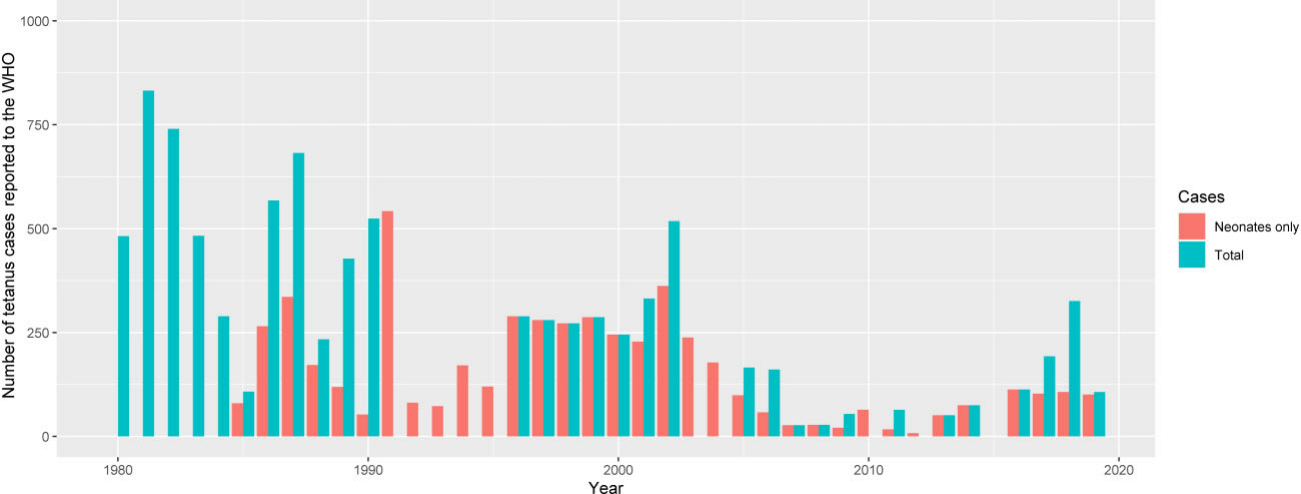
Total reported cases of tetanus in Guinea, 1980–2019.

The maternal and neonatal tetanus elimination (MNTE) initiative of the WHO launched in 1989 aimed to reduce the incidence of maternal and neonatal tetanus by 2015 to less than one case per thousand live births at district levels.^10^ Guinea was one of 59 countries initially identified under the MNTE initiative. Since 2010, there has been a further expansion of national policies against tetanus. The expansion includes implementation of the following measures: (1) vaccination of all women of childbearing age; (2) vaccination of pregnant women with two doses of tetanus toxoid (TT) vaccine; (3) promoting hygienic birth practices including improved umbilical cord care; and (4) vaccination of the infant with three doses of DPT vaccine. Guinea has also participated in global initiatives to meet the elimination target by implementing supplementary immunisation activities in high-risk regions.^11^ The raft of measures adopted has led to a steady reduction in the burden of neonatal tetanus.^12,13^ In 2017, the annual NNT incidence was 0.227 per 1000 live births (a total of 103 reported cases). Similarly, there has been a decline in overall incidence of tetanus from 35.59 per 100 000 people in 1990 to 3.96 per 100 000 in 2017.^14^ However, these rates are still higher than neighbouring countries: the corresponding incidence per 100 000 was 1.32 in Sierra Leone, 1.54 in Cote d'Ivoire, 3.18 in Guinea-Bissau and 3.05 in Mali.^14^ In 2019, the Guinean government launched three additional rounds of mass vaccination campaigns in all 38 health districts. The campaigns mobilised a workforce of 11 127 health workers and volunteers with a target to vaccinate over 3 million women of childbearing age.^9^ Despite these efforts to combat maternal and neonatal tetanus (MNT), the entire country is still considered to be at risk of MNT. At least one case of NNT has been reported in each of the 38 health districts during the preceding 5 y.^9^ Overall, a total of 193 tetanus cases were reported to the WHO in 2017, 326 in 2018 and 107 in 2019 (Figure 1).

The authors (IC, MEH, MD, ASD and FCon) have treated several cases of tetanus in the past few years while working in the paediatric ward of Kamsar Hospital, a referral hospital located in the Boké region in Western Guinea, which has below-national tetanus vaccine coverage during pregnancy.^12^ This work presents the results of an audit of all admissions to the neonatal and paediatric wards of the Kamsar Hospital in order to guide local interventions to eliminate the disease within the region.

## Material and Methods

### Study design

This is a retrospective audit of all admissions to the neonatal and general paediatric units of Kamsar Hospital from 1 January 2014 up to and including 31 December 2018. The aim of this study was to determine the incidence of NNT and PNNT among those admitted to the neonatal and general paediatric units of Kamsar Hospital.

### Study location

This study was conducted at Kamsar Hospital, which is located in the Boke region of Western Guinea. The port city of Kamsar lies ∼250 km north of the capital Conakry. In 2014, according to the population census, Kamsar had a population of 133 350, of whom 32% were children aged <10 y.^15^ Bauxite was discovered in the region in 1963 and this led to the development of Kamsar as a major mining city. The city has permanent access to electricity, running water and health services, including the well-equipped Kamsar Hospital. Kamsar Hospital is a major referral hospital in the region and admits patients from the entire Boké region, which has a population of just over a million. The hospital has an outpatient ward dedicated to consultation and an inpatient ward for hospitalisation along with intensive care units (ICUs). The hospital provides a dedicated paediatric service along with a general paediatric unit and a specialised neonatal unit. The paediatric ward has a capacity of 50 beds, including 12 beds for neonatal care. As of 2019, there were five paediatricians and several general physicians, as well as nurses and supporting staff providing care.

### Diagnosis of tetanus and patient management

The case definitions of NNT and PNNT were based on the WHO protocol.^16^ The Dakar prognostic scoring system was used to grade the severity of cases: mild (Dakar scores from 0 to 2), moderate (scores equal to 3) and severe (scores from 4 to 6).^1^ On admission, metronidazole and diazepam were administered to all patients. Tetanus immunoglobulin antitoxin was administered intramuscularly, and the following drugs were administered based on the judgement of treating clinicians: phenobarbital, electrolyte supplements and penicillin G. For severe cases, intensive care support was provided and the patients were kept on a ventilator with an oxygen extractor and a nasogastric tube for hydration, feeding and administration of oral medications.

### Data collection

The following patient characteristics were extracted: age, gender, body temperature, place of delivery of the newborn (home or hospital), suspected port of entry of the bacterium, incubation and onset period, complications encountered, requirement of nasogastric intubation support upon admission and the treatment regimen administered. Maternal characteristics such as education level, place of residence (rural/urban), number of antenatal care visits and vaccination against tetanus, including the number of doses received, were extracted. Information on clinical outcomes after treatment was extracted, including the length of hospital stay and mortality outcome.

### Statistical analyses

The descriptive characteristics of the included patients are presented. Survival probability was calculated using the Kaplan–Meier method. Univariable and multivariable Cox regression models were used to estimate the relative hazards of mortality within 7 d of admission. The results were reported following the RECORD (Reporting of studies Conducted using Observational Routinely collected Data) statement.^17^

## Results

From 1 January 2014 to 31 December 2018, there were a total of 5670 admissions to the neonatal and paediatric wards, of which 39 (0.7%) were due to tetanus (Table 1). Twenty-two (56.4%) cases were among neonates (aged <28 d), 1 (5.9%) was in an infant (aged ≥28 d to <1 y), 15 (38.5%) were among children aged 1 to <5 y and 1 (2.6%) was in a child aged 67 mo (Table 2). Among neonates (n=22), the entry site of the bacterium was identified as either the umbilical cord (n=20) or was circumcision-related (n=2). Among postneonatal cases (n=17), the entry sites were surface wounds in 12 (70.6%), limb fracture in 1 (5.9%) and the site could not be established in 4 (23.5%). Using the Dakar severity scale, there were 21 (53.8%) severe cases, 6 (15.4%) moderate cases and 12 (30.8%) mild cases.

**Table 1.** Number of hospital admissions and tetanus cases from 1^st^ Jan 2014 until 31^st^ Dec 2018 at paediatric and neonatal ward at Kamsar Hospital, Guinea

Year	Number of admissions to the paediatric and neonatal wards	Tetanus cases (%)
2014	853	12 (1.4%)
2015	1023	10 (1.0%)
2016	1202	7 (0.6%)
2017	1287	6 (0.5%)
2018	1305	4 (0.3%)
Overall	5670	39 (0.7%)

**Table 2.** Maternal and paediatric characteristics at enrolment

Characteristics	NNT	PNNT	Overall
Number of patients	22	17	39
Median age/d	7 [6–7; 1–28]	576 [432–576;192–2016]	13 [7–13;1–2016]
Median temperature/centigrade	39 [38–39;37–40]	39 [38–39;37–39]	39 [38–39;37–40]
Duration of onset of symptoms/d	3 [2–3;1–10]	10 [4–10;2–14]	4 [3–4;1–14]
Female	8 (36.4%)	6 (35.3%)	14 (35.9%)
Age group			
Neonate	22 (100.0%)	-	22 (56.4%)
Infant	-	1 (5.9%)	1 (2.6%)
1–59 mo	-	15 (88.2%)	15 (38.5%)
≥60 mo	-	1 (5.9%)^a^	1 (2.6%)
Tetanus entry location			
Postcircumcision	2 (9.1%)	0 (0.0%)	2 (5.1%)
Umbilical cord	20 (90.9%)	0 (0.0%)	20 (51.3%)
Wound	0 (0.0%)	12 (70.6%)	12 (30.8%)
Limb fracture	0 (0.0%)	1 (5.9%)	1 (2.6%)
Unknown	0 (0.0%)	4 (23.5%)	4 (10.3%)
Place of birth			
Home	9 (40.9%)	0 (0.0%)	9 (23.1%)
Hospital	13 (59.1%)	17 (100%)	30 (76.9%)
Dakar severity grading			
Mild	0 (0.0%)	12 (70.6%)	12 (30.8%)
Moderate	3 (13.6%)	3 (17.6%)	6 (15.4%)
Severe	19 (86.4%)	2 (11.8%)	21 (53.8%)
Place of residence			
Rural Kamsar	12 (54.5%)	5 (29.4%)	17 (43.6%)
Urban Kamsar	10 (45.5%)	12 (70.6%)	22 (56.4%)
Maternal education			
No formal education	22 (100.0%)	16 (94.1%)	38 (97.4%)
Number of antenatal care visits			
0	3 (13.6%)	5 (29.4%)	8 (20.5%)
1	10 (45.5%)	12 (70.6%)	22 (56.4%)
2	4 (18.2%)	0 (0.0%)	4 (10.3%)
3	5 (22.7%)	0 (0.0%)	5 (12.8%)
Maternal tetanus vaccination			
Unvaccinated	7 (31.8%)	10 (58.8%)	17 (43.6%)
Received 1 dose	12 (54.5%)	7 (41.2%)	19 (48.7%)
Received 2 doses	3 (13.6%)	0 (0.0%)	3 (7.7%)

Abbreviations: NNT, neonatal tetanus; PNNT, postneonatal tetanus.

Median values are presented with [IQR; range].

^a^ 67-mo-old

### Clinical signs and symptoms at presentation

The median duration for the onset of symptom was 4 d (IQR: 3–4 d; range: 1–14 d). The duration of symptom onset was 3 d (IQR: 2–3 d; range: 1–10 d) among neonates and 10 d (IQR: 4–10 d; range: 2–14 d) among postneonates. The overall common signs and symptoms at admission were dysphagia (92.3%), fever (89.7%), generalised muscle spasm (84.5%), trismus (79.5%) and heart rate >100 beats per min (64.1%) (Table 3). Trismus was present in all NNT cases and in only nine (52.9%) with PNNT.

**Table 3.** Signs and symptoms at presentation

Symptoms at presentation	Neonatal tetanus (N=22)	Postneonatal tetanus (N=17)	Overall (N=39)
Trismus (lockjaw)	22 (100.0%)	9 (52.9%)	31 (79.5%)
Dysphagia	20 (90.9%)	16 (94.1%)	36 (92.3%)
Paroxysm	13 (59.1%)	0 (0.0%)	13 (33.3%)
Respiratory difficulty	20 (90.9%)	0 (0.0%)	20 (51.3%)
Umbilical hernia	5 (22.7%)	0 (0.0%)	5 (12.8%)
Opisthotonus	11 (50.0%)	1 (5.9%)	12 (30.8%)
Generalised muscle spasm	20 (90.9%)	13 (76.5%)	33 (84.6%)
Heart rate >100 beats per min	19 (86.4%)	6 (35.3%)	25 (64.1%)
Shock	6 (27.3%)	1 (5.9%)	7 (17.9%)
Hypocalcaemia	0 (0.0%)	1 (5.9%)	1 (2.6%)
Temperature >37.5^o^C	19 (86.4%)	16 (94.1%)	35 (89.7%)

Abbreviation: N, number of patients.

### Baseline demographics

Seventeen (43.6%) tetanus cases were from rural areas. All the mothers of the patients in the NNT group had no formal education, while the corresponding proportion in the PNNT group was 94.1% (16/17). Overall, 17 (43.6%) children were born to mothers who did not receive a tetanus vaccination during pregnancy, 19 (48.7%) were born to mothers who had received one dose of tetanus vaccination and 3 (7.7%) were born to mothers who had received two doses (Table 2). Eight patients (20.5%) were born to mothers with no antenatal clinical visits.

### Survivorship

**Figure 2.**
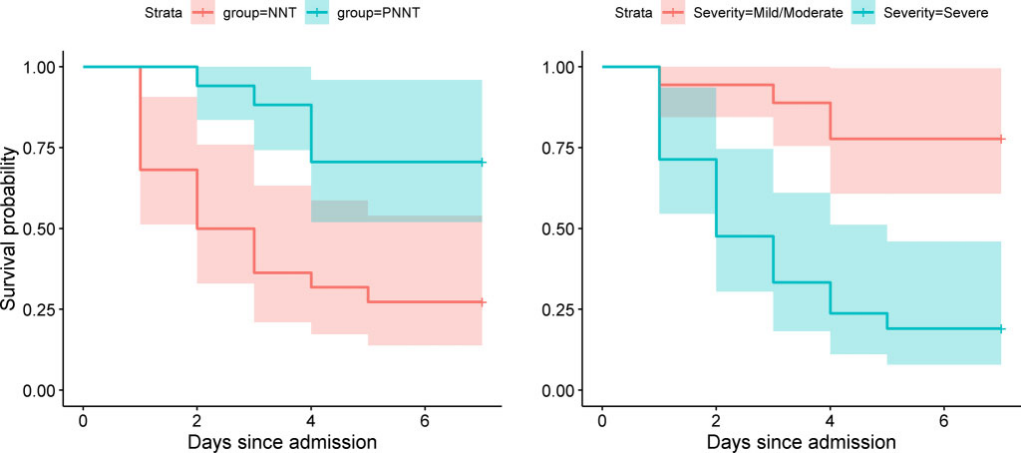
Kaplan-Meier estimate of survival probability within 7 d of hospital admission for (left panel) neonatal tetanus (NNT) and postneonatal tetanus (PNNT), and (right panel) disease severity graded using Dakar scoring rule. Log-rank test: p=0.003 for left panel and p<0.001 for right panel.

The median length of hospital stay was 2.5 d (IQR: 1–2.5 d; range: 1–36 d) for patients with NNT and 13 d (IQR: 4–13 d; range 2–43 d) in the PNNT group. There were 21 (53.8%) deaths (all deaths occurred within 7 d of hospitalisation); mortality within 7 d of hospitalisation was 72.7% (16/22) in the NNT group and 29.4% (5 /17) among those with PNNT (Figure 2; left panel). The Kaplan-Meier survivorship estimate was 0.222 (95% CI 0.066 to 0.754) among those born at home compared with 0.533 (95% CI 0.382 to 0.745) among those with hospital births (p=0.040; log-rank test). Survivorship decreased with increasing disease severity; the estimate was 0.778 (95% CI 0.608 to 0.996) among those with mild/moderate disease compared with 0.190 (95% CI 0.080 to 0.460) among those with severe disease (p<0.001; log-rank test) (Figure 2; right panel).

### Predictors of mortality

In univariable Cox regression, there were four predictors associated with mortality within 7 d of admission: homebirth (Hazards Ratio (HR): 2.55 [95% CI 1.02 to 6.35] compared with hospital birth); being a neonate (HR: 4.18 [95% CI 1.52 to 11.48] compared with non-neonates), admission to the ICU for resuscitation (HR: 5.58 [95% CI 2.24 to 13.88]) and presentation with severe disease (HR: 6.58 [95% CI 2.18 to 19.82]) compared with mild/moderate disease). In a multivariable regression analysis, none of the factors remained independently associated with mortality after adjusting for disease severity status (Table 4).

**Table 4.** Risk factors for mortality within 7 d of admission using Cox regression

Variable	N/n	Univariable HR [95% CI]	HR [95% CI] adjusted for disease severity
Maternal age/y	39/21	0.93 [0.86 to 1.02]	1.01 [0.91 to 1.12]
Tetanus group			
PNNT (reference)	17/5	1	1
NNT	22/16	4.18 [1.52 to 11.48]	1.16 [0.28 to 4.74]
Gender			
Female (reference)	14/8	1	1
Male	25/13	0.72 [0.30 to 1.75]	0.52 [0.21 to 1.28]
Place of birth			
Hospital (reference)	30/14	1	1
Home	9/7	2.55 [1.02 to 6.35]	1.00 [0.38 to 2.65]
Place of residence			
Urban Kamsar (reference)	22/11	1	1
Rural Kamsar	17/10	1.26 [0.53 to 2.97]	0.72 [0.29 to 1.78]
Maternal vaccination			
None (reference)	17/9	1	1
Fully/partially vaccinated	22/12	0.93 [0.39 to 2.22]	0.72 [0.29 to 1.76]
Admission to the ICU for resuscitation			
No (reference)	25/8	1	1
Yes	14/13	5.58 [2.24 to 13.88]	2.36 [0.76 to 7.35]
Severity of disease			
Mild/moderate (reference)	18/4	1	-
Severe	21/17	6.58 [2.19 to 19.82]	-

Abbreviations: CI, confidence interval; HR, hazards ratio; ICU, intensive care unit; N, total number of non-missing observations; n, number of deaths within 7 d of hospital admission; NNT, neonatal tetanus; PNNT, postneonatal tetanus.

## Discussion

Our audit identified 22 neonatal and 17 postneonatal patients admitted to the paediatric unit at Kamsar Hospital from 2014 to 2018. Nearly all (38/39) the babies were born to mothers with no formal education. A majority of the mothers were either unvaccinated or did not complete their two doses of TT immunisation scheduled during pregnancy. The suboptimal tetanus TT vaccination uptake during pregnancy is consistent with a previous report from the capital Conakry.^18^ However, it is alarming that >75% of the mothers had either never visited or only once attended antenatal care. Such suboptimal antenatal care utility reduces the likelihood of completing the TT immunisation and exposes the newborns to an additional risk of acquiring the disease. Of note, there were three cases of NNT despite the mothers receiving two doses of TT vaccination during pregnancy (all three neonates died; Table 2). The reasons for the three NNT cases developing in those born to vaccinated mothers remain unclear. The quality of the vaccine, potential degradation during transportation and poor storage facilities may be possible explanations.^19^ It could also possibly suggest interference with transplacental antibody transfer or could simply reflect natural variation in response as only 84% of neonates are estimated to be protected from tetanus by maternal vaccination.^4^

The umbilical cord was the bacterial entry site for most (20/22) of the NNT cases. Just over a third of the neonates were born at home (information on whether the birth was attended by a trained assistant was not known) and the remaining were born at a hospital. While homebirth might be associated with an increased risk of tetanus through contamination compared with facility-based deliveries, it was not possible to elucidate the reasons for 13 neonatal cases among those with hospital birth. Among those who were born at a hospital, the median age at admission to our hospital was 7 d (IQR: 6–7 d). It is possible that traditional practices believed to facilitate rapid healing of the umbilical stump were applied to these babies after they were discharged.^20^ For example, in 1996, Roisin et al. reported practices of applying *Karité* nut butter in Conakry, Guinea, and also remarked on the wide usage of similar products throughout West Africa.^6^ Chlorhexidine has already been adopted or is being adopted in over 25 countries globally as a part of umbilical cord care.^21^ It is currently not implemented in Guinea and when it is approved by the government, it will strengthen the cord care and reduce the likelihood of tetanus acquisition through the umbilical cord. There were also two cases of NNT that occurred following circumcision (both were from the rural Kamsar region). Information regarding the qualifications of the personnel who performed the circumcision was unavailable, but it is not uncommon for it to be performed by traditional practitioners, as reported in a study from the capital Conakry.^22^ Reports on NNT following circumcision remain rare in the literature, and the observation of two cases in our study suggests that the current elimination activities should also consider promoting safe circumcision practices.

The overall mortality within 7 d of admission was very high (53.8%), with a greater than 6-fold risk of death among those with severe disease relative to mild/moderate cases. Although the risk of mortality was also 4-fold higher in the NNT group than in the PNNT group in the univariable analysis, the difference was no longer apparent in a multivariable model that adjusted for severity status (Table 4). This strongly suggests that the higher mortality observed is likely attributable to disease severity at presentation rather than other patient characteristics. Just over half of the patients in our study had severe disease at presentation, which indicates an urgent need for the adoption of a standardised protocol for optimal patient management of severe cases to prevent case fatality. This further suggests that despite adequate hospital care, this disease remains difficult to treat. Taken together, our study suggests a strong need for raising awareness regarding maternal immunisation, encouraging optimal antenatal care and raising awareness of hygienic birth and circumcision practices.

Finally, our study also points towards the possibility of under-reporting of tetanus in the country despite it being a notifiable disease. For example, there were no officially reported cases of tetanus to the WHO in 2015, despite recording 10 cases at Kamsar Hospital (Figure 1, Table 1). This discrepancy could have arisen because Kamsar Hospital officially falls within the administration of the Ministry of Mining rather than the Ministry of Health. This indicates an urgent need for the integration of databases from different health service units to successfully eliminate the disease.

### Conclusions

In our setting, NNT and PNNT were rare causes of hospital admission, but when presented, the cases were often severe with a high mortality rate. The cases occurred primarily in children born at home to mothers who were either unvaccinated or inadequately vaccinated during pregnancy. Elimination efforts should focus on encouraging optimal antenatal care and raising awareness of maternal immunisation. These findings can help to design programmes to strengthen data surveillance systems and devise targeted interventions to eliminate NNT and PNNT in Guinea.

## Data Availability

The datasets used for current study are available from the Paediatrics unit at Kamsar Hospital University upon reasonable request. A request can be made to the corresponding author.
